# Computational Approach Improves Diagnostic Performance Based on Tumor-Associated Autoantibodies in Esophageal Squamous Cell Carcinoma

**DOI:** 10.34133/csbj.0025

**Published:** 2026-03-20

**Authors:** Yosui Nojima, Fumiaki Shiratori, Takashi Suzuki, Satoshi Yajima, Takeshi Toyozumi, Hideaki Shimada

**Affiliations:** ^1^ Center for Mathematical Modeling and Data Science, The University of Osaka, Toyonaka, Osaka 560-8531, Japan.; ^2^ Department of Computational Medicine, Nara Medical University, Kashihara, Nara 634-8524, Japan.; ^3^ Department of Surgery, School of Medicine, Toho University, Ota-ku, Tokyo 143-8541, Japan.; ^4^ Department of Frontier Surgery, Graduate School of Medicine, Chiba University, Chuo-ku, Chiba 260-8677, Japan.; ^5^ JCHO Funabashi Central Hospital, Funabashi, Chiba 273-8556, Japan.

## Abstract

Although tumor-associated autoantibodies (TAAbs) are promising biomarkers for early cancer detection, the diagnostic performance of individual TAAbs for esophageal squamous cell carcinoma (ESCC) remains limited. In this study, we constructed machine learning (ML) models for ESCC diagnosis using the serum levels of 6 TAAbs (c-myc, NY-ESO-1, p53, p62, RalA, and Sui1) and compared their performance with that of a conventional approach. When the training and test datasets were combined, serum samples from 339 patients with ESCC and 152 healthy controls were analyzed. To address class imbalance, the training data were oversampled using the synthetic minority oversampling technique. Classification models were developed using 5 ML algorithms, among which the svmLinear (support vector machine with a linear kernel), svmRadial (support vector machines with a radial basis function kernel), and neural network models demonstrated robust performance without overfitting. At specificities exceeding 90%, these models achieved substantially higher sensitivities than those obtained using any single TAAb. Notably, the svmLinear and neural network models achieved sensitivities greater than 40% for early-stage ESCC (stage 0/I) in the test dataset. Overall, the integration of multiple TAAbs with ML substantially improved the diagnostic performance for ESCC, highlighting the clinical potential of ML-based approaches.

## Graphical Abstract

**Figure:**
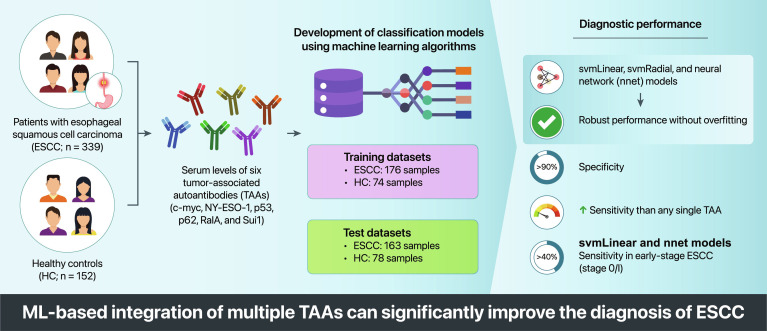

## Introduction

With the success of cancer immunotherapies represented by immune checkpoint inhibitors, the close relationship of cancer with the immune system from the earliest stages of its development has gained cognizance [1]. Tumor-associated autoantibodies (TAAbs) are produced as a result of the humoral immune response against cancer. In lung cancer, TAAbs can be detected in the serum up to 5 years before the definitive tumor diagnosis [2]. Moreover, TAAbs are reportedly detectable with higher sensitivity than tumor-associated antigens [3]. Serum anti-p53 antibody, a TAAb, is reimbursed under the Japanese national health insurance as an in vitro diagnostic test for esophageal, colorectal, and breast cancers and is currently used in clinical practice [4,5].

Patients with esophageal squamous cell carcinoma (ESCC) have a poor prognosis [6], despite attempts to control ESCC using multidisciplinary treatments [7]. In addition, the early detection of ESCC is an important treatment strategy for improving prognosis [8]. However, in ESCC, the sensitivity of anti-p53 antibodies alone is only 25%, and many patients with ESCC remain undiagnosed [9]. Moreover, no TAAbs with a diagnostic performance higher than that of anti-p53 antibodies have been identified. In our previous study, we evaluated the diagnostic performance of 6 TAAbs (c-myc, NY-ESO-1, p53, p62, RalA, and Sui1) using a conventional approach, which yielded sensitivities ranging from 7.8% to 30% [8], signifying no dramatic improvement in diagnostic performance. In recent ESCC-related studies, the application of machine learning (ML) techniques for early detection has increased in imaging-based research [10–12]. In contrast, studies integrating ML with TAAbs remain limited.

In this study, we constructed ML models for the diagnosis of ESCC using the serum levels of the 6 TAAbs described above in patients with ESCC and healthy controls (HCs) and compared their performance with that obtained using the conventional approach used in our previous study. Our results clarify the significance of ML approaches in the clinical diagnosis of ESCC.

## Materials and Methods

### Patients and sample collection

The number of patients and HCs in each cohort is shown in Table 1. This study analyzed 339 patients with histologically proven primary ESCC. The clinicopathological characteristics of patients from Chiba Cancer Center or those assigned to the test dataset have been reported in our previous studies [8,9]. Only the tumor–node–metastasis stage data were available for patients from BioBank Japan. The HCs had no history of malignant disease. The average age of the controls assigned to the training (*n* = 74) and test (*n* = 78) datasets was 37.9 and 48.2 years, and the male-to-female ratios were 35:39 and 49:29, respectively.

**Table 1.** Tumor-associated autoantibodies (TAAbs) used in this study

Dataset	Sample type	Cohort	Sample size	TAAbs					
				c-myc	Sui1	RalA	p62	p53	NY-ESO-1
Training	ESCC	BBJ	91	This study	This study	This study	This study	This study	This study
		CCC	85	This study	This study	This study	This study	Hoshino et al. [17]	Hoshino et al. [17]
	HC	MBL	74	Sumazaki et al. [26]					
Test	ESCC	KOU	26 (1)	Shiratori et al. [8] and this study					
		NUM	1 (1)						
		SCC	3 (0)						
		SUH	44 (8)						
		THO	30 (6)						
		TJU	4 (0)						
		TMD	49 (5)						
		TWM	6 (5)						
	HC	BBJ	78	Shiratori et al. [8]					

The numbers in parentheses indicate the number of samples newly utilized for this study out of the sample size of each cohort. “This study” refers to data newly used in the present research, whereas data cited from previous studies are reanalyzed data.

ESCC, esophageal squamous cell carcinoma; HC, healthy control; BBJ, BioBank Japan; CCC, Chiba Cancer Center; MBL, Medical & Biological Laboratories Co., Ltd.; KOU, Keio University School of Medicine; NUM, Nihon University School of Medicine; SCC, Saitama Cancer Center; SUH, Showa University School of Medicine; THO, Toho University School of Medicine; TJU, Jikei University School of Medicine; TMD, Tokyo Medical and Dental University; TWM, Tokyo Women’s Medical University.

Serum samples were collected before treatment and stored at −80 °C until use. All procedures were approved by the Research Ethics Boards of Toho University School of Medicine, Toho University Omori Medical Center (approval number: M23192), as well as by each participating hospital.

### Isolation, purification, and amplification of recombinant tumor-associated antigens and enzyme-linked immunosorbent assay of serum autoantibodies

Full-length cDNA of c-myc, p62, RalA, p53, Sui1, and NY-ESO-1 were amplified using the polymerase chain reaction. Briefly, the recombinant proteins were expressed in *Escherichia coli* BL21-CodonPlus (DE3)-RIL (Stratagene, La Jolla, CA, USA). Each TAAb extract was added to Ni Sepharose 6 Fast Flow (GE Healthcare UL, Buckinghamshire, UK), and the column was washed with 50 mM imidazole in phosphate-buffered saline. Purified recombinant tumor-associated antigens were eluted with 200 mM imidazole in phosphate-buffered saline. DNA sequencing confirmed that the correct gene had been inserted into the constructed plasmid. Serum samples collected from the patients and controls were analyzed using the enzyme-linked immunosorbent assay. As this test was conducted as a corporate clinical trial, its quality was approved by the Pharmaceuticals and Medical Devices Agency.

### Clustering and dimensionality reduction

To examine whether potentially concerning batch effects were present in the patient–TAAb matrix data, Gaussian mixture model (GMM) clustering was performed using the Mclust function of the mclust package (version 5.4.9) in R (version 4.2.3). The number of components was set as the number of cohorts when performing GMM clustering on the training and testing datasets. The patient–TAAb matrix data were subjected to dimensionality reduction using multidimensional scaling (MDS) and visualized in 2 dimensions with annotations indicating the results of GMM clustering and cohort information.

### ML model construction and evaluation

**Fig. 1.**
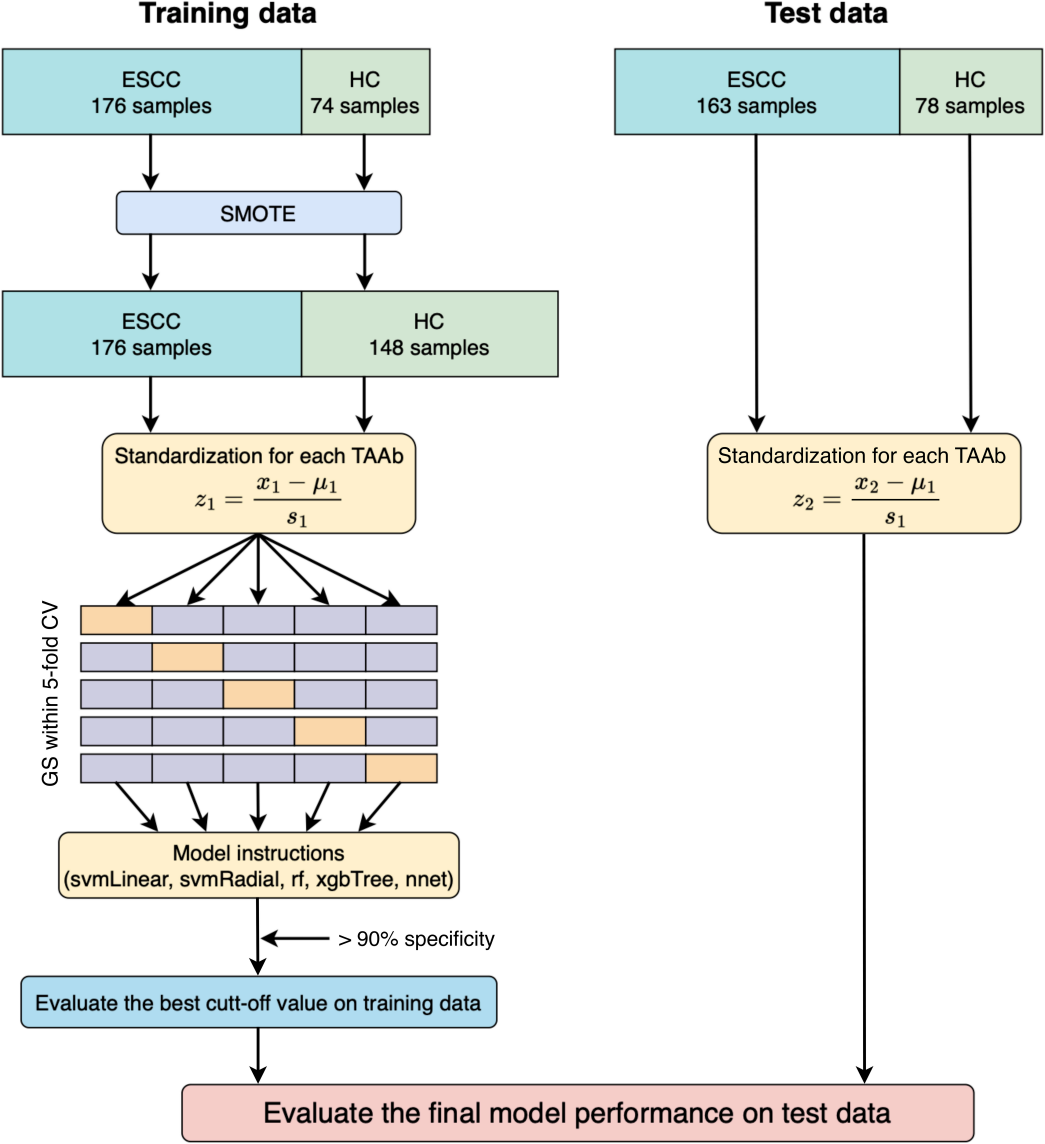
Overview of machine learning (ML) model construction and validation. Data on tumor-associated autoantibodies (TAAbs) for the training and testing datasets were obtained from multiple cohorts shown in Table 1. Training data imbalance was addressed using the synthetic minority oversampling technique (SMOTE), and models were constructed using 5 algorithms through 5-fold cross-validation and a grid search. Model accuracy was evaluated using test data. ESCC, esophageal squamous cell carcinoma; HC, healthy control; GS, grid search; CV, cross-validation.

The workflow for constructing the ML model is shown in Fig. 1. The training data were oversampled using the synthetic minority oversampling technique (SMOTE) [13] function of the DMwR package (version 0.4.1) in R. For the oversampled samples, the values of the 6 TAAbs were standardized for each patient using Eq. (1).

$$z_{1}=\frac{x_{1}-\mu_{1}}{\sigma_{1}}$$ (1)

where $z_{1}$ denotes the standardized TAAb values of the training dataset, $x_{1}$ denotes the TAAb value of the training dataset, $\mu_{1}$ denotes the mean value of each TAAb, and $\sigma_{1}$ denotes the sample variance. The test dataset was standardized using the mean and variance of the training dataset, assuming practical use in real-world clinical settings. Therefore, the values of the 6 TAAbs in the test dataset were standardized for each sample using Eq. (2).

$$z_{2}=\frac{x_{2}-\mu_{1}}{\sigma_{1}}$$ (2)

where $z_{2}$ denotes the standardized TAAb values of the test dataset and $x_{2}$ denotes the TAAb values in the test dataset.

Classification models were constructed using the training function of the caret package (version 6.0-94). The training samples were split into the training and validation sets in a ratio of 8:2, and model training was performed using 5-fold cross-validation. The support vector machines with a radial basis function kernel (svmRadial), support vector machine with a linear kernel (svmLinear), eXtreme gradient boosting (xgbTree), random forest (rf), and neural network (nnet) were selected to construct the classification models. The hyperparameter settings for each ML algorithm used in the grid search are listed in Table S1. When classifying normal controls and ESCC, probability thresholds were set in increments of 0.001, and the sensitivity and specificity were calculated at each threshold. The probability threshold that achieved a specificity of >90% in the training dataset was selected as the final threshold. The area under the curve (AUC) was calculated using the confusion matrix function of the caret package.

## Results

### Characterization of the TAAb datasets

**Fig. 2.**
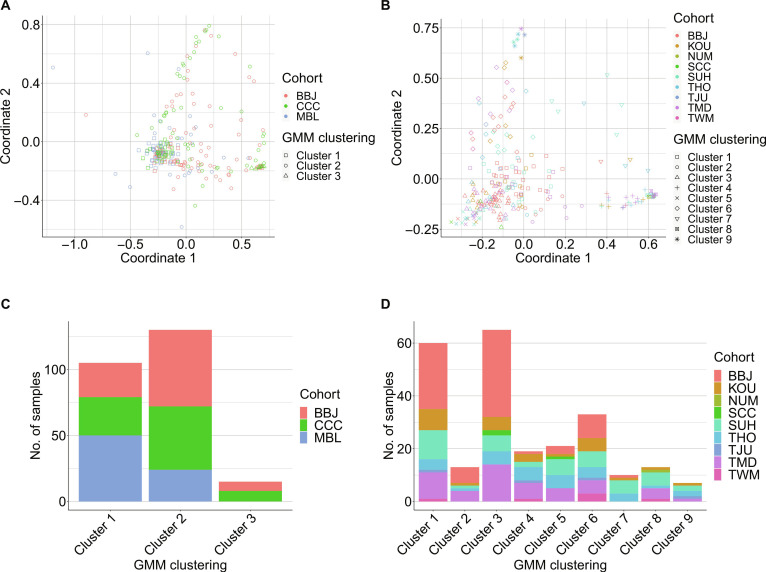
Multidimensional scaling (MDS) and Gaussian mixture model (GMM) clustering of the training (A) and test datasets (B). The plot colors indicate cohorts, and plot shapes represent GMM cluster assignments. The bar plots show the number of samples in each GMM-derived cluster for the training (C) and test datasets (D). BBJ, BioBank Japan; CCC, Chiba Cancer Center; MBL, Medical & Biological Laboratories Co. Ltd.; KOU, Keio University School of Medicine; NUM, Nihon University School of Medicine; SCC, Saitama Cancer Center; SUH, Showa University School of Medicine; THO, Toho University School of Medicine; TJU, Jikei University School of Medicine; TMD, Tokyo Medical and Dental University; TWM, Tokyo Women’s Medical University.

The serum samples used in this study were collected from 3 cohorts for the training data and 9 cohorts for the test data (Table 1). If a batch effect exists among the cohorts, the dataset cannot be directly used for training and must be corrected using an appropriate method. Therefore, we first examined the presence or absence of batch effects among the cohorts through visualization using MDS. MDS showed that clusters formed by specific cohorts were absent in the training and test datasets (Fig. 2A and B). We further examined the potential batch effects using GMM clustering and found that none of the detected clusters consisted of a single specific cohort, and no association was observed between the detected clusters and cohorts in either dataset (Fig. 2C and D). In addition, we generated violin plots for both the training and test datasets to examine the distribution within each cohort, confirming that no markedly different distributions were observed in either dataset (Fig. S1A and B). These results indicated the absence of a batch effect among the cohorts. Because the HCs differed in mean age between the training and test datasets, we performed correlation analyses between age and the serum level of each TAAb. For most TAAbs, the *P* values were greater than 0.05, indicating no significant correlation with age. Although a few TAAbs showed *P* values below 0.05, inspection of the scatter plots suggested that these findings were driven by outliers. Taken together, these results indicate that the serum levels of the 6 TAAbs evaluated in this study were not meaningfully influenced by age (Fig. S2A and B).

### ML model constructions and performance evaluations

Biomedical datasets are often severely imbalanced, rendering most ML algorithms unsuitable [14]. To address this problem, we used SMOTE to oversample the HCs in the training data. We then constructed classification models using the 5 algorithms to classify ESCC and HC. During the learning process, a grid search was performed to identify the optimal hyperparameters, followed by evaluation via 5-fold cross-validation (Fig. S3). The AUCs in each fold ranged from approximately 0.7 to 0.9, with no substantial differences among the ML models (Fig. S4).

**Fig. 3.**
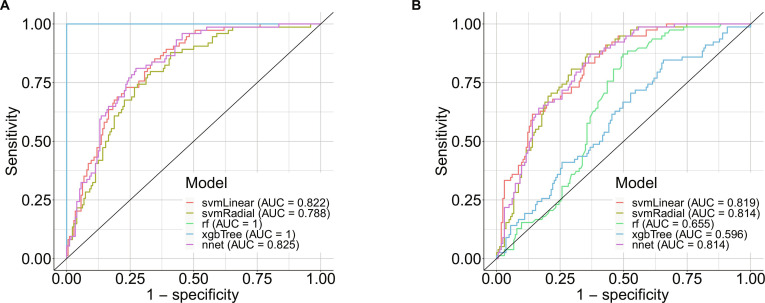
Model performance of each algorithm in the training (A) and test datasets (B). AUC, area under the curve.

At the >90% specificity cutoff in the training dataset, the ML models constructed with svmLinear, svmRadial, and nnet achieved sensitivities of 58.0%, 49.4%, and 57.4%, respectively, and specificities of 90.5% each in the training dataset (Table 2). For the test dataset, the 3 ML models achieved sensitivities of 52.8%, 44.8%, and 51.5% and specificities of 92.3%, 98.7%, and 92.3%, respectively. The sensitivity and specificity of the ML models constructed with rf and xgbTree in the training dataset were 100% and 90.5%, respectively. In contrast, their specificities in the test dataset plummeted to 0% and 8.97%, respectively. In the training dataset, the AUCs of svmLinear, svmRadial, and nnet were 0.822, 0.788, and 0.825, respectively, whereas those of rf and xgbTree were 1.0 and 1.0, respectively (Fig. 3A). In the test dataset, the AUCs of svmLinear, svmRadial, and nnet were 0.819, 0.814, and 0.814, respectively, whereas those of rf and xgbTree were 0.655 and 0.596, respectively (Fig. 3B). These results indicate that the models constructed with rf and xgbTree were overfitted, resulting in low performance on the test data. In contrast, the models built using svmLinear, svmRadial, and nnet were more robust, as evidenced by their high performances on the test data. Therefore, the ML models constructed using svmLinear, svmRadial, and nnet were used for the downstream analyses.

**Table 2.** Sensitivity and specificity in the training and test datasets at the optimal cutoff

	svmLinear		svmRadial		rf		xgbTree		nnet	
	Training	Test	Training	Test	Training	Test	Training	Test	Training	Test
Sensitivity	58.0% (50.3%–65.3%)	52.8% (44.8%–60.6%)	49.4% (41.8%–57.1%)	44.8% (37.0%–52.8%)	100% (97.9%–100%)	100% (97.8%–100%)	100% (97.9%–100%)	95.7% (91.4%–98.3%)	57.4% (49.7%–64.8%)	51.5% (43.6%–59.4%)
Specificity	90.5% (81.5%–96.1%)	92.3% (84.0%–97.1%)	90.5% (81.5%–96.1%)	98.7% (93.1%–99.9%)	90.5% (81.5%–96.1%)	0% (0%–4.62%)	90.5% (81.5%–96.1%)	8.97% (3.69%–17.6%)	90.5% (81.5%–96.1%)	92.3% (84.0%–97.1%)

Values in parentheses indicate the 95% confidence intervals.

### Differences in sensitivities among stages and cohorts

The sensitivities for each stage in the training and test datasets for the 3 ML models (svmLinear, svmRadial, and nnet) are listed in Table 3. In the training dataset, the sensitivities of svmLinear and nnet were identical at 45.5% and 59.6% for stages 0/I and III, respectively. For stage II, svmLinear yielded a sensitivity of 55.3%, whereas nnet showed a sensitivity of 70.5% for stage IV, which were the highest among the models for these respective stages (Table 3). The sensitivities of svmRadial were lower than those of the svmLinear and nnet at all stages. In the test dataset, svmLinear showed the highest sensitivity for all stages: 41.6% for stage 0/I, 62.5% for stage II, 60.5% for stage III, and 73.3% for stage IV (Table 3). In addition, the sensitivities of the ML models were higher in the advanced stages than in the early stages (Table 3). To evaluate the robustness of the models, we calculated sensitivities using leave-one-cohort-out cross-validation (LOCOCV), taking into account the limited sample sizes in some cohorts. LOCOCV demonstrated that comparable sensitivities were obtained across all combinations in which each cohort was excluded in turn (Table 4).

**Table 3.** Sensitivities for each stage and each model in the training and test datasets

Dataset	Model	Stage			
		0/I	II	III	IV
Training	svmLinear	45.5% (28.1%–63.6%)	55.3% (38.3%–71.4%)	59.6% (45.8%–72.4%)	68.2% (52.4%–81.4%)
	svmRadial	33.3% (18.0%–51.8%)	42.1% (26.3%–59.2%)	52.6% (39.0%–66.0%)	63.6% (47.8%–77.6%)
	nnet	45.5% (28.1%–63.6%)	50.0% (33.4%–66.6%)	59.6% (45.8%–72.4%)	70.5% (54.8%–83.2%)
Test	svmLinear	41.6% (30.4%–53.4%)	62.5% (40.6%–81.2%)	60.5% (44.4%–75.0%)	73.3% (44.9%–92.2%)
	svmRadial	35.1% (24.5%–46.8%)	58.3% (36.6%–77.9%)	46.5% (31.2%–62.3%)	66.7% (38.4%–88.2%)
	nnet	41.6% (30.4%–53.4%)	62.5% (40.6%–81.2%)	58.1% (42.1%–73.0%)	66.7% (38.4%–88.2%)

Values in parentheses indicate the 95% confidence intervals.

Number of samples in the training dataset: stage 0/I (*n* = 33), stage II (*n* = 38), stage III (*n* = 57), and stage IV (*n* = 44).

Number of samples in the test dataset: stage 0/I (*n* = 77), stage II (*n* = 24), stage III (*n* = 43), and stage IV (*n* = 15).

**Table 4.** Sensitivities in leave-one-cohort-out cross-validation (LOCOCV)

Model	Excluded cohort							
	THO	KOU	SUH	TWM	TMD	SCC	TJU	NUM
svmLinear	53.4% (44.5%–62.1%)	55.5% (46.7%–64.0%)	52.1% (42.8%–61.3%)	51.6% (43.5%–59.6%)	54.4% (44.8%–63.7%)	51.9% (43.8%–59.8%)	51.6% (43.5%–59.6%)	52.5% (44.5%–60.4%)
svmRadial	43.6% (35.0%–52.5%)	48.2% (39.6%–56.9%)	43.7% (34.6%–53.1%)	43.9% (36.0%–52.1%)	47.4% (37.9%–56.9%)	43.8% (35.9%–51.8%)	44.0% (36.2%–52.1%)	44.4% (36.6%–52.4%)
nnet	49.6% (40.8%–58.4%)	54.7% (46.0%–63.3%)	51.3% (41.9%–60.5%)	50.3% (42.2%–58.4%)	54.4% (44.8%–63.7%)	50.6% (42.6%–58.6%)	50.9% (42.9%–58.9%)	51.2% (43.3%–59.2%)

Values in parentheses indicate the 95% confidence intervals.

THO, Toho University School of Medicine; KOU, Keio University School of Medicine; SUH, Showa University School of Medicine; TWM, Tokyo Women’s Medical University; TMD, Tokyo Medical and Dental University; SCC, Saitama Cancer Center; TJU, Jikei University School of Medicine; NUM, Nihon University School of Medicine.

## Discussion

In this study, we constructed 5 ML models to diagnose ESCC or HCs using the serum levels of 6 TAAbs as independent variables. Among these, 3 models (svmLinear, svmRadial, and nnet) achieved higher sensitivities and specificities for the training and test datasets than those in our previous study. For individual TAAbs, the maximum sensitivity without distinguishing stages was approximately 30% [8]. In contrast, the present models outperformed any individual TAAb in terms of sensitivity. In particular, the sensitivity for identifying stage 0/I in the test dataset exceeded 40% with the svmLinear and nnet models. Thus, the ML approach using TAAbs improves the diagnostic performance for ESCC. In addition, the consistent performance of our models in LOCOCV suggests their ability to maintain diagnostic accuracy across multiple medical institutions.

Li et al. [15] developed an ML model that diagnosed ESCC using 5 TAAbs (CAST, HDAC1, HSF1, PTMS, and ZPR1). The final performance in the test set included a sensitivity of 66.7% and 73.8% in the early and advanced stages, respectively, and a specificity of 87%. Xu et al. [16] constructed a model for the same purpose using 4 TAAbs (LDOA, ENO1, p53, and NY-ESO-1). The final performance in external set 2 included a sensitivity of 67.3% or 61.4% in the early stage and a specificity of 90.1% or 95.0%. Because there is generally a trade-off between sensitivity and specificity, improving one usually compromises the other. However, the ML model constructed by Xu et al. [16] showed higher sensitivity and specificity compared with the model constructed by Li et al. [15], indicating that the model developed by the former had better performance. In this study, we built ML models for the diagnosis of ESCC using 6 TAAbs (c-myc, NY-ESO-1, p53, p62, RalA, and Sui1). Therefore, p53 and NY-ESO-1 overlapped with the independent variables used in the ML model of Xu et al. [16], suggesting that autoantibodies against p53 and NY-ESO-1 are important for predicting ESCC using ML models.

The p53 gene is mutated in most solid cancers, leading to frequent mutations and abnormal expression of the p53 gene and protein in these diseases [17]. The generation of p53 autoantibodies is hypothesized to be related to p53 protein overexpression, and missense mutations in the TP53 gene lead to the loss of protein function [16,18,19]. NY-ESO-1 is another commonly studied autoantibody. A previous study suggested that the NY-ESO-1 antigen elicits humoral immune responses not only by mutant but also by wild-type epitopes [16,20]. In our previous studies, we reported the presence of p53 autoantibodies in many cancer types [17,21,22]. In addition, a high positive rate of the NY-ESO-1 autoantibody has been reported not only in esophageal cancer but also in other cancer types [17,23–25]. Furthermore, we previously showed that serum levels of these TAAbs were higher in patients with hepatocellular carcinoma (HCC) or breast cancer than in HCs [26,27]. Therefore, autoantibodies against p53 and NY-ESO-1 may be useful for improving the diagnostic performance of ML models, not only for ESCC but also for other cancer types.

Although the autoantibodies against c-myc, p62, RalA, and Sui1 did not overlap with the independent variables used in the ML models built by Li et al. [15] and Xu et al. [16], their serum levels in patients with ESCC and HCC were higher than those in HCs [8,27]. In breast cancer, the serum levels of autoantibodies against RalA and Sui1 are higher than those in HCs, whereas those of autoantibodies against c-myc and p62 are not elevated [26]. Therefore, when constructing ML models for cancer diagnosis, autoantibodies against c-myc, p62, RalA, and Sui1 may be effective for specific cancer types, rather than being broadly effective across various cancers.

It is easier to construct ML models with a high diagnostic performance for ESCC because the pathological differences between ESCC and normal tissue are relatively clear. In contrast, most patients with HCC experience progression from a normal liver to chronic hepatitis (CH) and liver cirrhosis (LC) before tumorigenesis. Therefore, diagnostic models must not only distinguish between normal liver tissue and HCC but also between HCC and CH or LC. Moreover, from a clinical perspective, distinguishing HCC from CH or LC is more meaningful than distinguishing normal from HCC. Nevertheless, Wu et al. [28] measured various TAAbs in the serum of HCs and patients with HCC or LC and constructed diagnostic ML models. The diagnostic performance was relatively high for distinguishing normal tissue from HCC but not sufficiently high for distinguishing HCC from LC. Thus, despite its clinical importance, the construction of a practical ML model that accurately distinguishes HCC from LC (and CH) is considered highly challenging. Wu et al. [28] did not use autoantibodies against c-myc, NY-ESO-1, p53, p62, RalA, or Sui1 as independent variables when constructing their ML models. Further studies are warranted because the utility of autoantibodies against these antigens in building ML models to distinguish HCC from LC or HCC from CH remains unclear.

## Conclusion

**Fig. 4.**
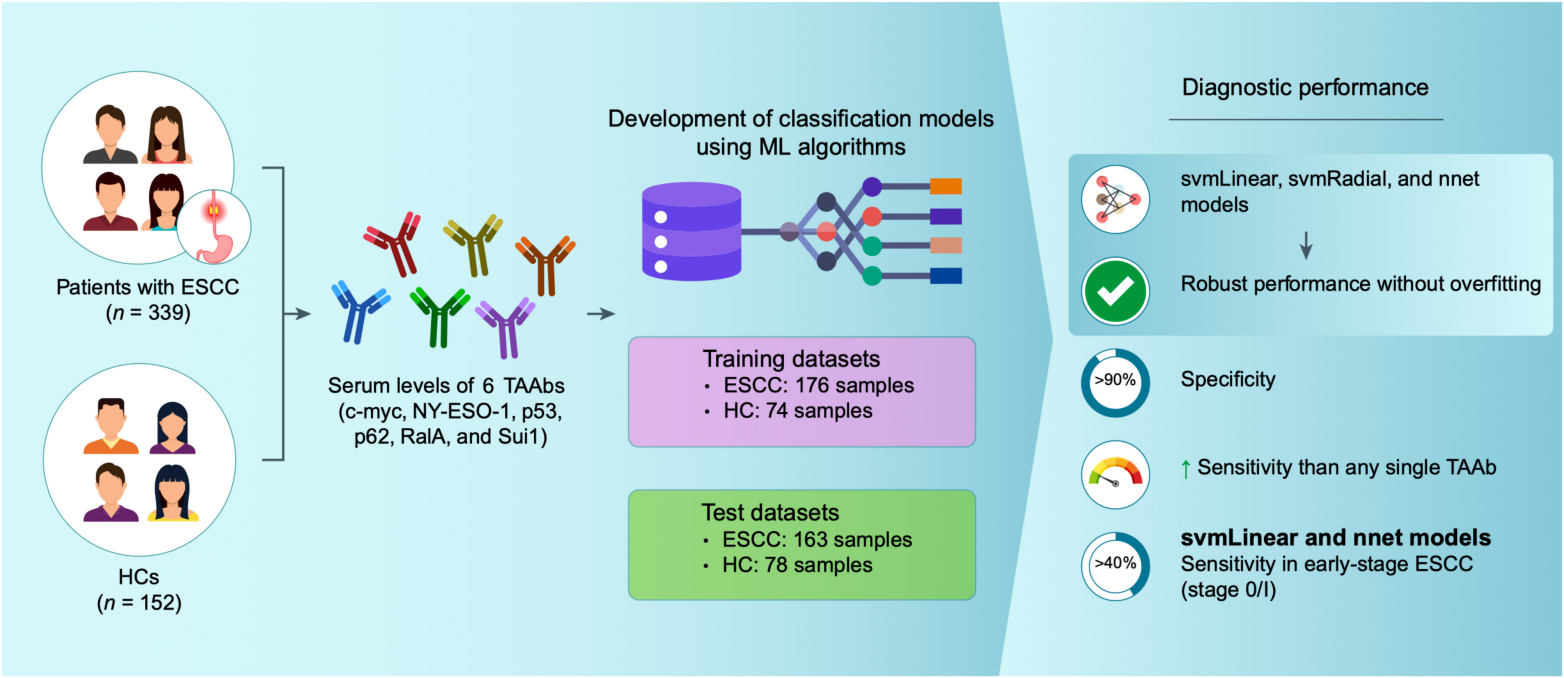
Overview of the study design and conclusions. TAAbs, tumor-associated autoantibodies; ESCC, esophageal squamous cell carcinoma; HCs, healthy controls; ML, machine learning.

In this study, we developed and evaluated ML-based diagnostic models for ESCC using the serum levels of 6 TAAbs. Compared with conventional single-marker approaches, the ML models, particularly those based on svmLinear, svmRadial, and nnet, demonstrated substantially improved diagnostic performance with robust generalizability across independent test datasets. Notably, these models achieved sensitivities exceeding 50% at high specificity cutoffs (>90%), representing a marked improvement over the limited diagnostic performance of individual TAAbs such as anti-p53 antibodies alone. Importantly, enhanced performance was also observed in early-stage ESCC, where early detection is clinically critical yet remains challenging using existing biomarkers (Fig. 4).

Overall, our findings demonstrate that the ML-based integration of multiple TAAbs offers a promising and practical strategy for improving the serological diagnosis of ESCC. This approach may complement the existing diagnostic modalities and has the potential to be extended to other cancer types, warranting further validation in prospective and clinically diverse cohorts.

## Data Availability

All data supporting the findings of this study are available within the paper and the Supplementary Materials. All analyses were performed using the cited software, packages, and pipelines, whose codes are publicly available. Further information about the code used in this study can be obtained from the corresponding author on reasonable request.
