# Supplementary material for: Computational Approach Improves Diagnostic Performance Based on Tumor-Associated Autoantibodies in Esophageal Squamous Cell Carcinoma
Source: Comput Struct Biotechnol J. 2026 Mar 20;35(1):0025. doi: 10.34133/csbj.0025 (PMC13068007; doi:10.34133/csbj.0025)
Supplement: Supplementary 1 — Figs. S1 to S4 Table S1 [file csbj.0025.f1.pdf]

Supplementary Materials

Computational approach improves diagnostic performance based on tumor-associated autoantibodies in esophageal squamous cell carcinoma

Yosui Nojima[a,b*], Fumiaki Shiratori[c], Takashi Suzuki[c], Satoshi Yajima[c], Takeshi Toyozumi[d], Hideaki Shimada[c,e]

[a]Center for Mathematical Modeling and Data Science, The University of Osaka, 1-3 Machikaneyama, Toyonaka, Osaka 560-8531, Japan

[b]Department of Computational Medicine, Nara Medical University, 88 Shijo-cho, Kashihara, Nara 634-8524, Japan

[c]Department of Surgery, School of Medicine, Toho University, 6-11-1 Omori-nishi, Ota-ku, Tokyo 143-8541, Japan

[d]Department of Frontier Surgery, Graduate School of Medicine, Chiba University, 1-8-1 Inohana, Chuo-ku, Chiba 260-8677, Japan

[e]JCHO Funabashi Central Hospital, 6-13-10 Kaijin, Funabashi, Chiba 273-8556, Japan

[*]Corresponding author

To whom correspondence should be addressed:
Yosui Nojima, Ph.D.
Center for Mathematical Modeling and Data Science, Osaka University
1-3 Machikaneyama, Toyonaka, Osaka 560-8531, Japan
Tel: +81-66-850-6091 / Fax: +81-66-850-6092
E-mail: nojima.yosui.mmds@osaka-u.ac.jp

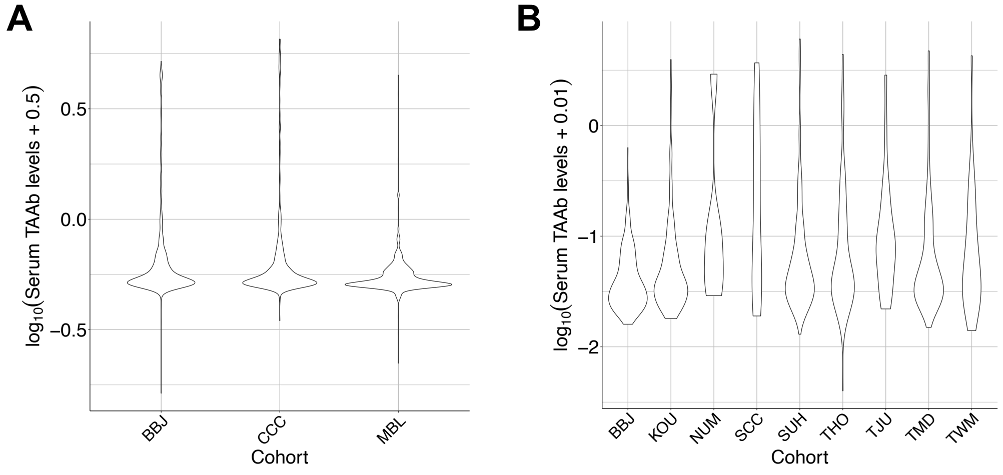

Fig. S1. Distribution of each cohort in the training (A) and test (B) datasets. To ensure that all values were positive before log transformation, an offset of 0.5 was added to the training set and 0.01 to the test set, respectively.

BBJ, BioBank Japan; CCC, Chiba Cancer Center; MBL, Medical & Biological Laboratories Co., Ltd.; KOU, Keio University School of Medicine; NUM, Nihon University School of Medicine; SCC, Saitama Cancer Center; SUH, Showa University School of Medicine; THO, Toho University School of Medicine; TJU, Jikei University School of Medicine; TMD, Tokyo Medical and Dental University; TWM, Tokyo Women's Medical University

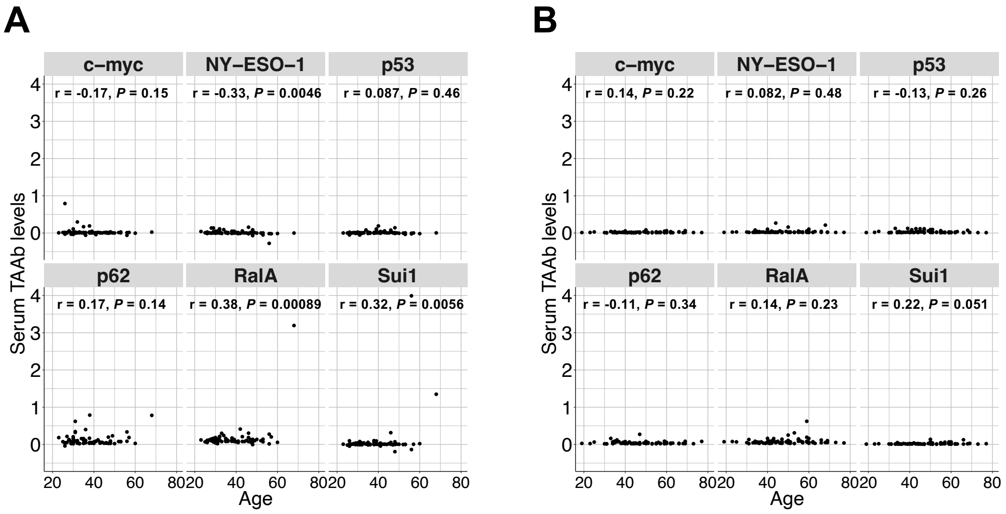

Fig. S2. Correlation analyses between age and serum tumor-associated autoantibody levels in healthy controls from the training (A) and test (B) datasets.

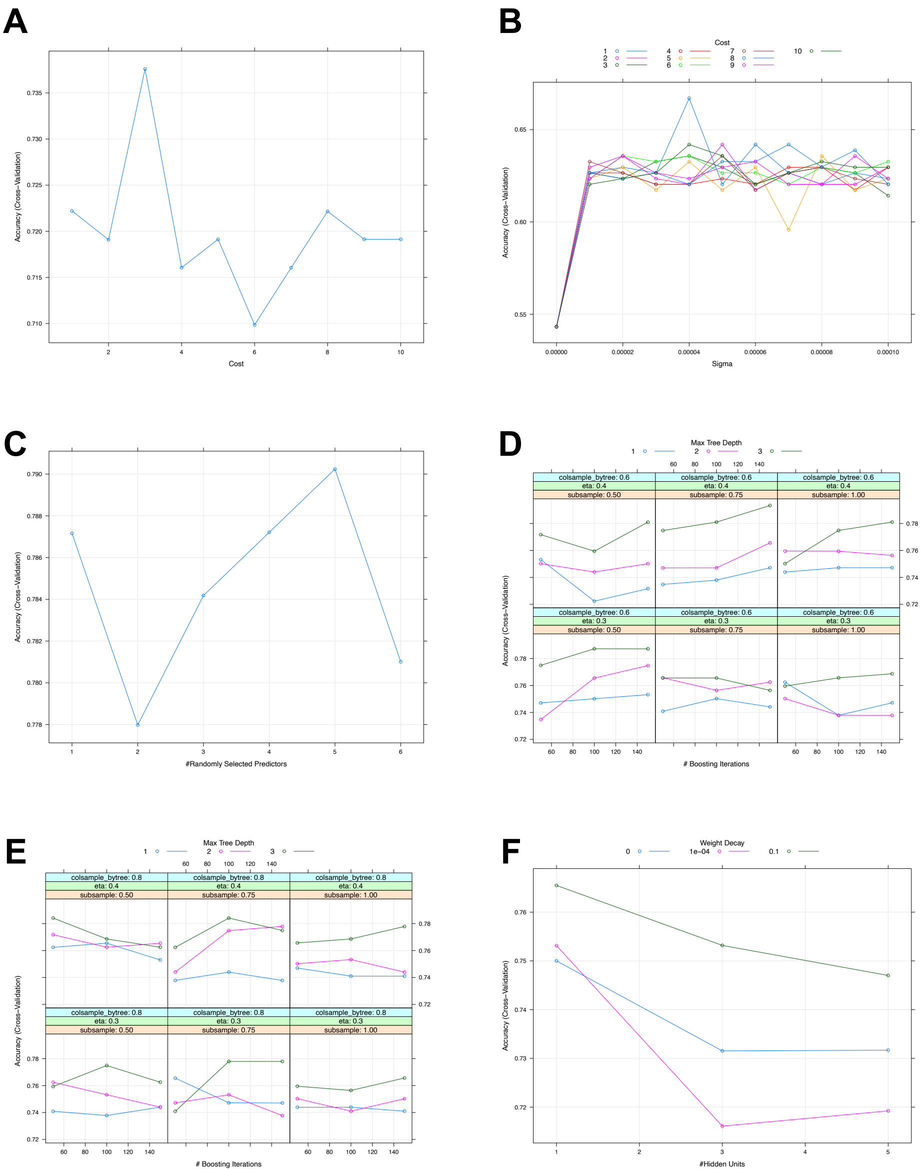

Fig. S3. Accuracy of each hyperparameter setting during grid search cross-validation. (A) support vector machines (SVM) with radial basis function kernel (svmRadial), (B) SVM with linear kernel (svmLinear), (C) random forest (rf), (D and E) eXtreme gradient boosting (xgbTree), (F) neural network (nnet).

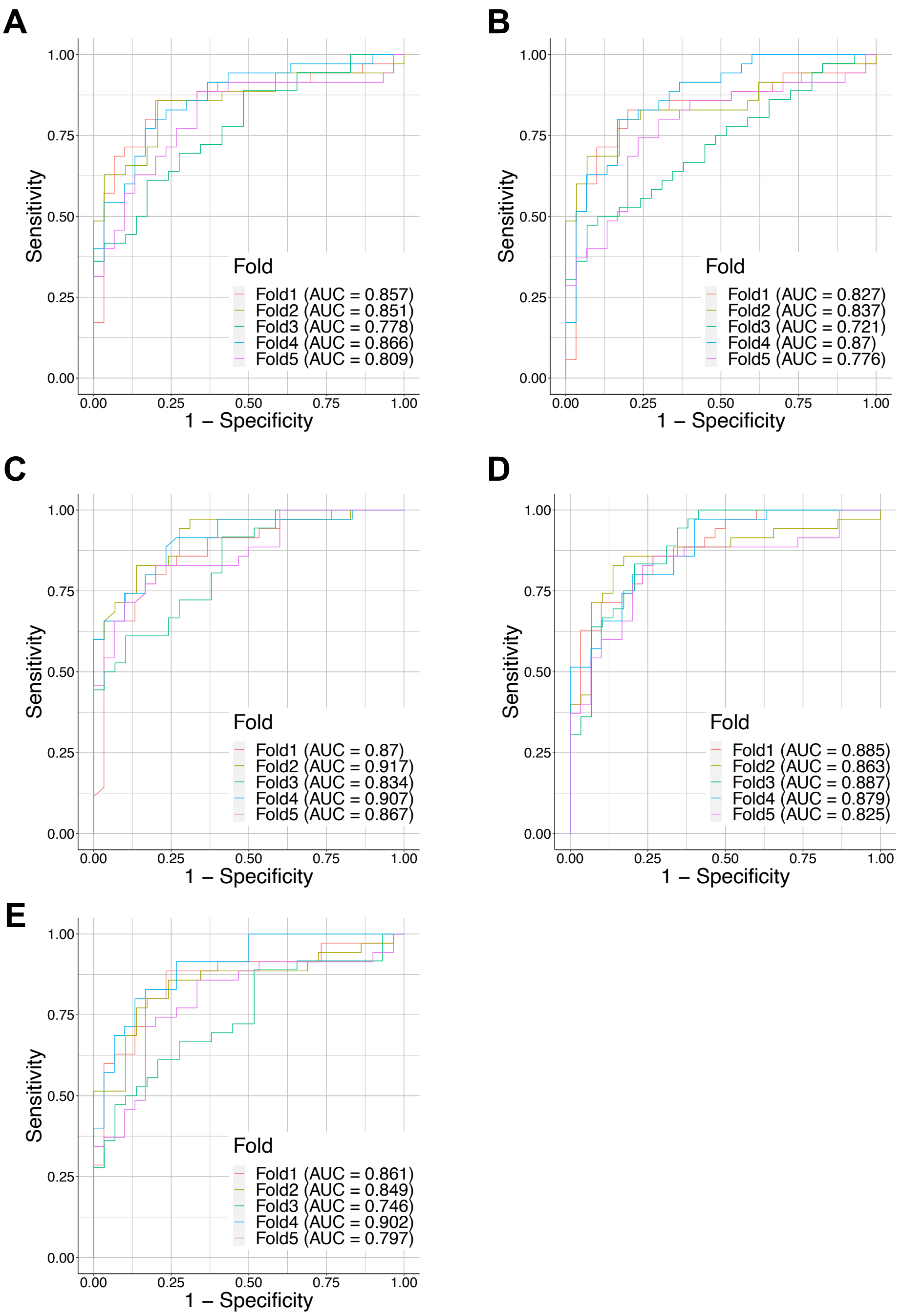

Fig. S4. Receiver operating characteristic curve and the area under the curve (AUC) of each fold in 5-fold cross-validation. **(A)** SVM with linear kernel (svmLinear), **(B)** support vector machines (SVM) with radial basis function kernel (svmRadial), **(C)** random forest (rf), **(D)** eXtreme gradient boosting (xgbTree), **(E)** neural network (nnet).

Table S1. Hyperparameter settings for each machine learning algorithm in the grid search.

svmLinear	svmRadial	xgbTree	rf	nnet
C = 1–10 in 1 increment	C = 1–10 in 1 increment<br>sigma = 0–0.0001 in 0.00001 increment	Default setting	mtry = 1–6 in 1 increment	Default setting
